# Receptor-like leucine-rich repeat kinases of subfamily III are involved in the recognition of Pectobacterium spp. by Solanaceae plants

**DOI:** 10.18699/vjgb-25-58

**Published:** 2025-07

**Authors:** E.V. Shrub, N.V. Kalubaka, P.V. Vychyk, O.A. Badalyan, Y.A. Nikolaichik

**Affiliations:** Belarusian State University, Minsk, Belarus; Belarusian State University, Minsk, Belarus; Belarusian State University, Minsk, Belarus; Belarusian State University, Minsk, Belarus; Belarusian State University, Minsk, Belarus

**Keywords:** receptor-like protein kinase, Solanaceae, Pectobacterium, effector, plant immunity, рецепторподобные протеинкиназы, Solanaceae, Pectobacterium, эффектор, растительный иммунитет

## Abstract

The genomes of Solanaceae plants contain over 600 receptor-like protein kinase genes with leucine-rich repeats (LRR-RLK), many likely associated with pathogen detection, but very few functionally characterized. Pectobacterium spp. are the major bacterial pathogens of agricultural crops, particularly potatoes and other Solanaceae plants. For relevant potato pathogens from the genus Pectobacterium, specific immune receptors have not been described in Solanaceae. However, in Malus × domestica, four LRR-RLK from the LRRIII subfamily (DIPM1-4) have been characterized as receptors for the related pathogen Erwinia amylovora. DIPMs specifically interact with the effector protein DspE and are involved in E. amylovora recognition. Since the DspE ortholog is also the main effector in Pectobacterium spp., we performed a phylogenetic analysis of LRRIII subfamily receptors in the most relevant Solanaceae representatives together with a much better characterized LRR-RLKIII of Arabidopsis thaliana and identified nine clusters of related RLKs. Clustering followed by analysis of published data allowed us to functionally characterize this RLK family and suggest the most likely candidates for checking interactions with the main effector of pectobacteria, DspE. Testing the kinase domains of representative cluster members in a yeast two-hybrid system revealed four Solanaceae RLKs interacting with the DspE effector from Pectobacterium versatile. Virus-induced silencing of these RLK genes demonstrated their involvement in P. versatile recognition. The RLK6 gene from Solanum bulbocastanum, which is not an ortholog of the DIPM proteins in apple, seems to be the most promising potential resistance gene. This work expands our understanding of LRR-RLKIII subfamily RLKs and their role in plant immunity, providing a foundation for future development of disease-resistant Solanaceae varieties.

## Abstract (ru)

Геномы растений семейства Пасленовые содержат более 600 генов рецепторных протеинкиназ с лейцин-богатыми повторами (LRR-RLK), многие из которых, вероятно, связаны с детекцией патогенов, но лишь некоторые были функционально охарактеризованы. Энтеробактерии рода Pectobacterium – основные бактериальные патогены многих сельскохозяйственных культур, в том числе картофеля и других растений семейства Пасленовые. Для актуальных патогенов из рода Pectobacterium специфические иммунные рецепторы растений не описаны. Однако у Malus × domestica охарактеризовано четыре LRR-RLK из подсемейства LRRIII (DIPM1-4), специфически взаимодействующих с эффекторным белком DspE и участвующих в распознавании родственного энтеробактериального фитопатогена Erwinia amylovora. Поскольку ортолог DspE является основным эффектором и у Pectobacterium spp., мы выполнили филогенетический анализ RLK-LRRIII растений семейства Пасленовые совместно с более полно охарактеризованными LRR-RLKIII у Arabidopsis thaliana и выделили девять кластеров родственных RLK. Кластеризация и анализ опубликованных данных позволили функционально охарактеризовать это семейство RLK и предложить наиболее вероятных кандидатов для проверки взаимодействия с основным эффектором пектобактерий DspE. Тестирование киназных доменов репрезентативных представителей разных кластеров в дрожжевой двухгибридной системе выявило четыре RLK растений семейства Пасленовые, которые взаимодействуют с эффектором DspE из Pectobacterium versatile (Pve). Уровень экспрессии генов этих RLK и их ортологов у разных растений семейства варьировал, но в целом был очень низким. При этом обнаружена сильная DspE-зависимая супрессия генов RLK2 и RLK5 у инфицированных Pve растений картофеля, а инактивация их ортологов предотвращала развитие сверхчувствительной реакции в листьях растений, инфильтрованных суспензиями Pve. Данная работа расширяет понимание разнообразия RLK подсемейства LRR-RLKIII и их роли в иммунитете растений и может способствовать селекции устойчивых к бактериозам сортов растений семейства Пасленовые.

## Introduction

Pectobacterium spp. are major bacterial pathogens of a range of important crops, particularly potatoes. P. atrosepticum, P. carotovorum, P. parmentieri, P. brasiliense, and P. versatile are the most relevant pathogenic species for potato. Depending on conditions and strain characteristics, pectobacteria can infect underground or above-ground parts of the plant, causing soft rot of tubers, blackleg, or aerial stem rot. The hallmark of Pectobacterium infections is the massive production of around 30 exoenzymes (pectolytic, cellulolytic, and proteolytic), leading to the characteristic softening of infected tissues (Chatterjee et al., 1995; Pérombelon, 2002).

Most potato varieties are susceptible to Pectobacterium infections. Although relatively tolerant varieties are known (Kwenda et al., 2016), potato plants resistant to Pectobacterium have not yet been developed. This situation can be largely explained by an insufficient understanding of the mechanisms by which plants recognize these pathogens, especially at the earliest stage of the infection.

Pectobacterium spp. were long considered typical necrotrophs minimally interacting with their hosts. However, since the advent of the genomic era, a substantial amount of data has accumulated, indicating the complex nature of molecular communication between Pectobacterium spp. and their hosts. A notable feature of this communication is the ability of Pectobacterium to coexist with its hosts in a “stealth” mode – without developing a systemic infection and without causing significant damage to plant tissues (Toth, Birch, 2005; Gorshkov et al., 2018).

The switch between these two fundamentally different phases of infection, latent and symptomatic, depends on regulatory events that are still poorly understood, particularly the early stages of plant-pathogen interaction. For instance, the pathogen’s quorum sensing system activates the synthesis of numerous virulence factors only at high population densities (Liu H. et al., 2008). The specialized repressor of pectin degradation, KdgR, is inactivated by the products of polygalacturonate hydrolysis (Liu Y. et al., 1999; Skoblyakov et al., 2004), which occurs only after significant cell wall degradation. Additionally, the PhoPQ-dependent switch in the pathogen’s metabolism and transmembrane transport is triggered by the release of divalent cations during cell wall degradation (Kravchenko et al., 2021). These three regulatory mechanisms are well studied but operate relatively late in the infection process; by this time some damage to the plant is already inevitable, making them less effective as targets for pathogen control. Therefore, the initial stage of pathogen recognition by the plant, mediated by membrane receptors, appears more promising. However, specific plant receptors for Pectobacterium spp. have not yet been described.

The classical model of plant immunity (Jones, Dangl, 2006) is based on the specific recognition of pathogenassociated molecular patterns (PAMP/MAMP, Pathogen/ Microbe-Associated Molecular Pattern) and effectors, which induce interconnected pathways of PAMP-triggered immunity (PTI) and effector-triggered immunity (ETI). PAMPs are often conserved proteins (flagellin, translation elongation factor Tu) (Gómez-Gómez, Boller, 2000; Zipfel et al., 2006), lipids (e. g., 3-hydroxydecanoic acid) (Kutschera et al., 2019), peptidoglycan (Willmann et al., 2011), and polysaccharides (Kawaharada et al., 2015).

Effectors are proteins translocated by the pathogen directly into plant cells. Some effectors, such as Avr proteins of the fungal pathogen Cladosporium fulvum (Fulvia fulva), act outside the cell (Rooney et al., 2005). The primary function of effector proteins is to disrupt plant signaling pathways responsible for pathogen recognition and immune response activation, with effector mechanisms being quite diverse (Giraldo, Valent, 2013; Macho, Zipfel, 2015; Zhang S. et al., 2022). Despite the key role of effectors in pathogen adaptation to its host, the presence of a plant immune receptor specific to a particular effector (encoded by an R-gene) activates ETI and provides resistance to infection.

PAMP/MAMP detection is carried out by membrane receptor complexes, while effector receptors triggering ETI can be either cytoplasmic or membrane-bound (Böhm et al., 2014; Couto, Zipfel, 2016; Bentham et al., 2020; Sun, Zhang J., 2020). Membrane receptors for MAMP/PAMP and cytoplasmic receptors for effectors can physically interact with each other (Qi et al., 2011). Despite different initial components, the subsequent signaling pathways and activated immune responses largely overlap, so the difference between PTI/MTI and ETI is quantitative rather than qualitative (Navarro et al., 2004; Thomma et al., 2011; Yuan et al., 2021).

Receptor complexes typically include co-receptors, which can be part of many receptor complexes. Receptors and coreceptors belong to several protein families, but most are part of the leucine-rich repeat (LRR) receptor domain family (Shiu, Bleecker, 2003; Chakraborty et al., 2019; Dievart et al., 2020). Receptors usually have more than 10 LRRs, while coreceptors have fewer than 9. Specific receptors may or may not have a cytoplasmic kinase domain and are called receptor-like kinases (RLK) or receptor-like proteins (RLP). Co-receptors typically have a kinase domain and can phosphorylate other components of the receptor complex, including the receptor itself. A receptor complex may include several co-receptors (mandatory in receptor complexes involving RLP) (Huang, Joosten, 2025).

Applying the classical zigzag model of immunity (Jones, Dangl, 2006) to Pectobacterium is challenging due to the limited data on PAMP-triggered responses (Kröner et al., 2011; Kuzmich et al., 2014), and the fact that, to date, only one effector protein, DspE (DspA), has been identified for Pectobacterium spp. (Nikolaichik et al., 2005; Kim J.-G. et al., 2011). DspE belongs to the AvrE superfamily of type III secretion system (T3SS) effectors (Nikolaichik et al., 2005; Degrave et al., 2015). Effectors in this family are named in different species after “avirulence”, “disease-specific protein” and “water-soaking” according to phenotype induced in plants (e. g. AvrE, DspE, and WtsE) and are considered critical for pathogens with a small number of effectors (Erwinia spp., Pantoea spp., and Pectobacterium spp.) (Gaudriault et al., 1997; Frederick et al., 2001; Mor et al., 2001; Kim H.-S. et al., 2011).

DspE of P. versatile (Pve) is required for successful infection of the host plant. It is also the main inducer of the hypersensitive response (a typical sign of ETI) in non-hosts. DspE is delivered into plant cells via T3SS and can be detected within host cells as early as 3 hours after infection with a noninduced culture (Nikolaichik et al., 2005). DspE triggers local and systemic defense responses, indicating the plant’s ability to detect this effector protein (Nikolaichik, 2009).

The first direct evidence of a plant’s ability to specifically recognize DspE was obtained by studying the ortholog of this effector from Erwinia amylovora, the causative agent of apple fire blight. In yeast two-hybrid screen, four receptor-like kinases (DIPM1-4) were identified in Malus × domestica plants, the intracellular domains of which specifically interacted with DspE (Meng et al., 2006). DIPMs (DspE-Interacting Proteins from Malus) are receptor-like kinases with leucine-rich repeats in the sensory domain (LRR-RLK), belonging to the third subfamily (LRR-RLKIII).

Inactivation of certain DIPMs via silencing or genome editing increased plant resistance to fire blight (Borejsza-Wysocka, 2006; Pompili et al., 2020). A similar approach allowed us to identify three receptor-like kinases in tomato and tobacco that interact with DspE from P. versatile. Silencing the RLK2 and RLK5 genes in Nicotiana benthamiana reduced the plant’s ability to recognize P. versatile, leading to a weakened hypersensitive response (Nikolaichik et al., 2012; Badalyan, Nikolaichik, 2014). Three receptor-like kinases (WIP3-5) that specifically interact with WtsE, an ortholog of DspE from Pantoea stewartii subsp. stewartii, were also identified in Zea mays, but their functions have not yet been studied in planta (Jin et al., 2016).

The phenotype of apple and N. benthamiana plants with inactivated DIPM1-4 and RLK2/5 indicates that these receptor- like protein kinases are responsible for plant sensitivity to DspE-producing pathogens, meaning they can be considered S-genes. Inactivation of S-genes can be used to create resistant plants. However, the characterized DspE-interacting kinases partially duplicate each other’s functions, and their full spectrum is unknown, so complete elimination of pathogen sensitivity through the inactivation of a single or even a couple of these receptor genes seems unlikely. On the other hand, to ensure resistance to the pathogen, a single “suitable” R-gene might be sufficient, and such a gene encoding an LRR-RLKIII has been described for another pathosystem (Zhao et al., 2019).

The discussion above shows that LRR-RLKIII can be candidates for S- and R-genes against various pathogens and can also perform functions related to plant growth and development. This work summarizes and classifies available data on LRR-RLKIII with a focus on plants of the Solanaceae family, and may simplify the search for promising resistance genes for use in breeding programs of Solanaceae plants. We also provide an example of using this LRR-RLKIII classification to identify a potential resistance gene to pectobacterial infection.

## Materials and methods

Plant material and microorganism strains. Solanum tuberosum cv. Ragneda and N. benthamiana plants were grown in non-sterile nutrient soil at 20 °C with a 16-hour photoperiod. The following microbial strains were used: P. versatile JN42 (mcrB::ISPcc2, ΔfliTEFG, CmR (Tn9), RifR), VKE (JN42 dspE) (Nikolaichik et al., 2005); A. tumefaciens GV3101 (RifR, GmR, vir+) (Arabidopsis Biological Resource Center); Saccharomyces cerevisiae SKY48 (MATα, trp1, his3, ura3, lexAop-LEU2, cIop-LYS2), SKY473 (MATa, his3, leu2, trp1, ura3, lexAop-LEU2, cIop-LYS2) (Serebriiskii et al., 2005). The JN42 strain is derived from the natural P. versatile isolate 3-2, which lacks flagella due to a deletion within the fli cluster (GenBank CP024842). Cultures of P. versatile and A. tumefaciens were grown on LB medium, while S. cerevisiae was cultured on YPD medium at 28 °C.

Nucleic acids. The following plasmids were used: pTRV2, p1039, p1044, p1046 (Liu Y. et al., 2002), obtained from the Arabidopsis Biological Resource Center; pTRV2::RLK2, pTRV2::RLK5; pJG4-5; pJG4-5::dspF, pJK202::dspE (Nikolaichik et al., 2012); pJG4-5::ʹslRLK2, pJG4-5::ʹslRLK5, pJG4-5::ʹntRLK5 (Badalyan, Nikolaichik, 2014). The oligonucleotide sequences for RT-qPCR are listed in Table S1 (Supplementary Materials)1.


Supplementary Materials are available in the online version of the paper: https://vavilov.elpub.ru/jour/manager/files/Suppl_Shrub_Engl_29_4.pdf


Molecular cloning. A fragment of the C00T013379 (sbRLK6) gene from S. bulbocastanum was amplified using the primers 5ʹ-ccgaattcggtttatttcctggtaagat-3ʹ and 5ʹ-cgcctcga gggccaactcattgagaatcag-3ʹ and cloned into the pJG4-5 and pTRV2 vectors using EcoRI and XhoI restriction sites.

Protein-protein interaction analysis. Protein-protein interactions were analyzed using the LexA-based yeast twohybrid system (Serebriiskii et al., 2007). The bait plasmid had a fragment of the dspE gene cloned into the pJK202 vector. For positive control of interaction with DspE, the secretory chaperone DspF, specific to DspE, was used (Valentovich et al., 2008). Cells of the S. cerevisiae SKY473 strain were transformed with pJG4-5 derivatives. Cells of the opposite mating type SKY48 were transformed with pJK202::dspE. To detect protein-protein interactions, diploid S. cerevisiae cells obtained by crossing SKY473 and SKY48 strains were cultured on a selective medium for 2–4 days, followed by a “blue-white” test for β-galactosidase activity.

Protein sequence analysis. Genomic assemblies and annotations of the following versions were used: S. tuberosum DM v.6.1 (Pham et al., 2020), S. bulbocastanum (Tang et al., 2022), S. lycopersicum cv. Micro-Tom v.1.2.1 (Kudo et al., 2017), Arabidopsis thaliana Araport11 as of 2022-09-14 (Cheng et al., 2017).

Identification of receptor-like kinases in proteomes and their classification into families was performed using iTAK v. 1.2 (Zheng et al., 2016). Redundancy in sequences was reduced using the easy-cluster algorithm of the MMseqs2 program (Steinegger, Söding, 2017). For the alignment of kinase domain amino acid sequences, the MAFFT web service was used (Rozewicki et al., 2019). Alignment correction was performed using Jalview 2.11.2.7 (Waterhouse et al., 2009). A maximum likelihood dendrogram was constructed using ITREE v. 2.1.3 based on the Edge-linked partition model (von Haeseler et al., 2014; Chernomor et al., 2016), and graphical visualization was performed using the iTOL 6.8 service (Letunic, Bork, 2021).

Virus-induced gene silencing (VIGS). VIGS in N. benthamiana plants was performed using the TRV2 vector as described by Y. Liu et al. (2002). The hypersensitivity test was conducted 40 days after VIGS induction by infiltrating plant leaves with suspensions of P. versatile strains in 0.85 % NaCl at a density of 1.5 × 108 cells/ml using a needle-less syringe. At least 10 plants of each type were subjected to infiltration with all types of suspensions and NaCl solution as a control. For each type of suspension or NaCl solution, two or more leaves per plant were used. Results were recorded and samples were collected 24 hours after infection.

RT-qPCR. Leaf samples from N. benthamiana were collected 24 hours after infiltration (as described above) with P. versatile cell suspensions. Potato tubers were inoculated with an automatic pipette using suspensions of the same density (1.5 × 108 cells/ml) in a volume of 10 μl. Potato tissue samples were collected at the maceration zone boundary 48 hours after inoculation. RNA extraction and RT-qPCR were performed in six biological replicates as described (Nikolaichik et al., 2009). Gene expression levels were determined by RT-qPCR relative to the reference genes CAC, EF1A, TBP for N. benthamiana, and SAND, CAC, EF1a for S. tuberosum and calculated with the REST2009 2.0.13 software (Qiagen, USA).

## Results

**Phylogenetic analysis identifies distinct structural and functional subgroups within LRR-RLKII**I

Since all known RLKs (Receptor-Like Kinases) that interact with AvrE-like effectors belong to the LRR-RLKIII family, we analyzed the spectrum of RLKs in this family across various members of the Solanaceae family and compared them with the characterized LRR-RLKIII (mostly from A. thaliana). The iTAK classifier assigns between 45 and 77 RLKs to LRR-RLKIII in different Solanaceae species and 47 RLKs in A. thaliana (see the Table), leaving many options for identifying potential receptors involved in immunity regulation and pathogen detection, including P. versatile

**Table 1.**
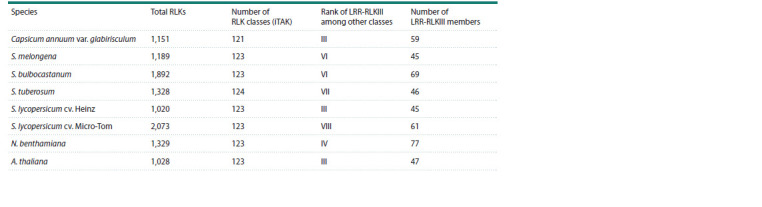
Quantitative analysis of RLKs in plants of the Solanaceae family

**Fig. 1.**
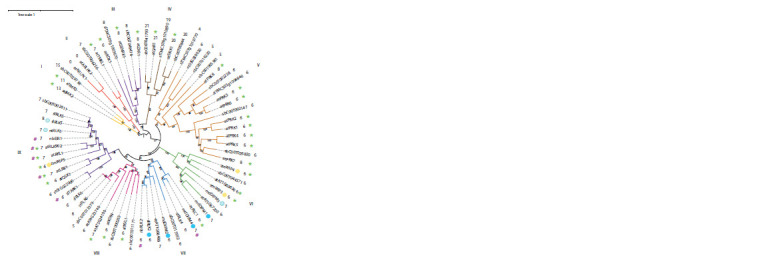
Dendrogram of the LRR-RLKIII family members. Only kinase domain sequences were used. Species are indicated by two-letter codes: at – A. thaliana, md – Malus × domestica, nb – N. benthamiana, nt – N. tabacum, sb – S. bulbocastanum, sl – S. lycopersicum, st – S. tuberosum, zm – Z. mays. Clusters of similar RLKs are highlighted in color and labeled with Roman numerals. LRR-RLKs interacting with AvrE family effectors (DspA/E, WtsE) are marked with circles, where the shading intensity is proportional to the interaction strength, and the color (blue or yellow) indicates whether the blue-white test was performed. Numbers next to the identifiers show the number of leucine-rich repeats (LRR) within the sensory domain. Experimentally studied LRR-RLKs are marked with “*” if they are involved in growth and development regulation and/or “#” if they are involved in immunity control.

Phylogenetic analysis allowed us to identify nine clusters of related LRR-RLKIII (Fig. 1). For RLKs in clusters I–V and VIII, published information does not show a connection to immunity. In the remaining three clusters (VI, VII, and IX), kinases capable of binding AvrE-like effector proteins are present but immunity-related functions have only been demonstrated for members of clusters VII and IX. More detailed information on experimentally characterized LRR-RLKIII is provided in Table S2.

**Identification of a NEw DspE-interacting LRR-RLKIII in S.**

So far, no R-genes have been identified among those encoding DspE-interacting RLKs. Most known immunity-related genes from this family, such as mdRLK4, slTARK1, nbEIR1, and ntRLK2, can be classified as S-genes. However, at least one clear R-gene in this family (RLK902 of A. thaliana, required for resistance to Hyaloperonospora arabidopsidis) has been described (ten Hove et al., 2011), suggesting the possibility of identifying resistance genes to other pathogens in this family. RLKs with a demonstrated role in immunity (including those interacting with AvrE-type effectors) are present only in clusters VII and IX. The connection to immunity of DspE/WtsE-interacting RLKs from cluster VI has not yet been shown but seems plausible. Cluster VIII is the closest to clusters VII, IX, and VI, but none of its members was shown to interact with DspE. Therefore, we selected a representative from cluster VIII for experimental analysis: the C00T013379 gene from S. bulbocastanum, a species often used as a source of R-genes suitable for potato resistance breeding.

**Fig. 2.**
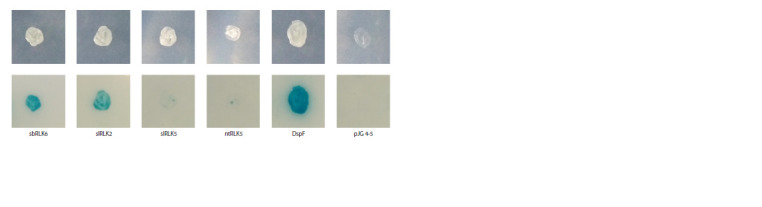
Interaction of the effector protein DspE with kinase domains of RLKs. Growth of diploids on leucine-deficient medium with X-gal. All cells contain plasmids pJK202-DspE, pDR8 with lacZ, and derivatives of plasmid pJG4-5 with an insertion of the reading frame of the indicated gene.

The cloned fragment of C00T013379, encoding the cytoplasmic part of the RLK, gave a positive result in the interaction test with DspE in the yeast two-hybrid system (Fig. 2). Based on the intensity of growth and coloration of yeast macro-colonies on selective medium, the interaction of DspE with kinase domain amplified from S. bulbocastanum was at the level of slRLK2 and the positive control (DspF) and significantly exceeded the interaction intensity with slRLK5 and ntRLK5 (orthologs of nbEIR1). By analogy with the previously characterized LRR-RLKIII, we designated this gene as sbRLK6.

**Fig. 3.**
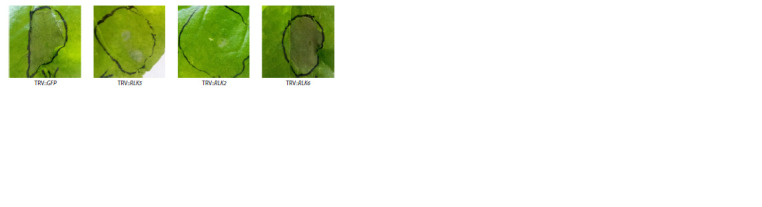
Hypersensitive response of N. benthamiana plants subjected to RLK gene silencing. Control plants were infected with TRV containing a neutral insert (GFP).

Silencing the ortholog of sbRLK6 in N. benthamiana, unlike silencing nbRLK2 and nbRLK5, did not affect the intensity of the hypersensitive response upon infiltration of leaves with Pve JN42 cell suspension (Fig. 3).

**P. versatile suppresses plant LRR-RLK genes**

**Fig. 4.**
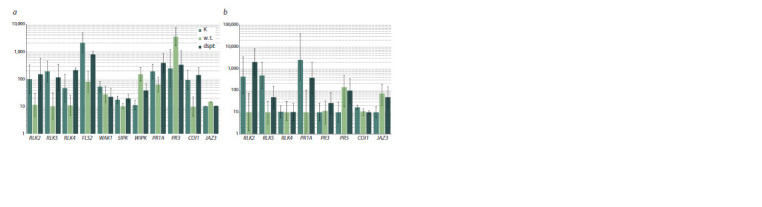
Changes in gene expression levels in N. benthamiana (a) and S. tuberosum (b) plants inoculated with suspensions of wild-type P. versatile (w. t.) and dspE mutant (dspE) or 0.85 % NaCl solution (K). Average values (in arbitrary units) of six measurements with 95 % confidence intervals are shown.

To understand the consequences of DspE recognition by LRRRLKIII, we assessed the expression levels of key immunity marker genes in both host plants (S. tuberosum) and non-host plants (N. benthamiana). Radical changes were observed for genes involved in salicylic acid and jasmonic acid signaling, as well as for the RLK genes (Fig. 4).

In Pve-infected N. benthamiana plants, expression of salicylic acid-dependent genes PR1A and SIPK was significantly reduced (Fig. 4a). In contrast, the jasmonic acid-dependent transcriptional activator gene JAZ3 was induced, while COI1 encoding the inhibitor of this pathway was repressed, and the marker genes WIPK and PR3 were strongly induced. Importantly, both suppression and induction of these genes were dependent on DspE, as the response of plants to inoculation with dspE mutant bacteria was much weaker. Similar DspEdependent suppression of the salicylic acid pathway marker PR1A was observed in Pve-infected potato plants (Fig. 4b). COI1 was slightly repressed, while JAZ3 – strongly induced, but the effect was independent of DspE.

Expression of RLK2 and RLK5 orthologs in both plants, as well as that of RLK4 in N. benthamiana, was also reduced in a DspE-dependent manner (Fig. 4a, b). The very low expression level of stRLK6 did not allow for an assessment of its change

To see how typical the observed expression pattern is during Pve infection, we checked the expression of two well-studied RLK genes from other families in N. benthamiana: FLS2, encoding the flagellin receptor, and WAK1, encoding a cell wall-associated kinase involved in oligogalacturonate perception. FLS2 responded to contact with wild-type Pve and the dspE mutant similarly to RLK2 and RLK5 (Fig. 4a). WAK1 in Pve-infected plants showed a barely noticeable (approximately twofold) but reproducible suppression, independent of DspE.

## Discussion

The phylogenetic analysis allowed us to divide LRR-RLKIII into two distinct functional groups. According to the available information, we conclude that members of clusters I–V are primarily involved in controlling plant growth and development, while clusters VI–IX are enriched with RLKs associated with the regulation of immune responses. Most LRR-RLKIII, including all those interacting with DspE, lack a key aspartate residue in the conserved catalytic loop motif (HRDXXXXN) and are therefore classified as pseudokinases. However, many pseudokinases retain some kinase activity and can function as part of receptor complexes containing an active kinase (Rodriguez-Furlan et al., 2022). Additionally, LRR-RLKIII generally have a small number (5–7) of leucine-rich repeats in their sensor domain, meaning they can only perform their signaling function as part of complex receptor systems that must include two additional critical components: a kinase and a receptor (or receptor-like protein) with a full set (more than 10, typically around 20) of leucine-rich repeats.

Known DspE-interacting proteins belong to clusters VI, VII, and IX. Among these, the role in pathogen recognition has been established for four proteins: mdDIPM4 and nbRLK2 from cluster VII and ntRLK5 and nbEIR1 from cluster IX. In all these cases, suppression of the RLK gene increased plant resistance (Borejsza-Wysocka et al., 2006; Nikolaichik et al., 2012; Badalyan, Nikolaichik, 2014; Pompili et al., 2020). Based on these results, mdDIPM4, nbRLK2, and nbEIR1 can be considered S-genes. However, we note that due to the cross-regulation of RLK genes reported in both apple (Borejsza-Wysocka et al., 2006) and tobacco (Badalyan, Nikolaichik, 2014), the observed phenotype cannot be unequivocally linked to the suppressed gene. Another member of cluster IX, TARK1, is also a product of an S-gene, as its over-expression enhances, while its inactivation weakens disease symptoms (Kim J.-G. et al., 2009; Campos, 2020; Guzman et al., 2020).

The sbRLK6 gene, described for the first time in this study, encodes an LRR-RLKIII belonging to cluster VIII, where no DspE-interacting RLKs had been previously described. Silencing the ortholog of sbRLK6 in N. benthamiana plants did not (unlike silencing RLK2 and RLK5) weaken the hypersensitive response of plants upon contact with Pve bacteria. For necrotrophs (including Pve), necrosis accompanying the hypersensitive response is favorable for expanding the infection zone and further colonization of the plant, so we classify RLK2 and RLK5 as S-genes. However, sbRLK6 cannot currently be considered an S-gene. The question of whether RLK6 can function as an R-gene requires its stable inactivation or over-expression followed by testing plant resistance when infecting organs (tubers and stems) that are typical targets of pectobacteria but are poorly suited for the virus-induced gene silencing technology used here.

Since the genes of co-receptor-like RLK2, RLK5, and the newly described RLK6 exhibit an expression pattern during Pve infection similar to that of the pattern recognition receptor gene FLS2, and the expression of all four genes shows signs of common control through the salicylic acid signaling pathway, we can hypothesize that DspE-interacting LRR-RLKIII are components of complex receptor systems involved in detecting Pve. Other components of such complexes and the cytoplasmic signaling proteins interacting with them may be considered promising candidates for identifying resistance genes to Pve and other bacteria

## Conclusion

So far, specific resistance genes to Pectobacterium spp. have not been described, and targeted breeding of potatoes for resistance to pectobacteriosis is not being conducted, not least due to the lack of information on R-genes that could provide such resistance. We propose to use receptor protein kinases of the RLK-LRRIII family, which specifically recognize the main effector protein of pectobacteria, DspE, for this purpose. As a first step in this direction, this study has compiled information on experimentally studied members of this family, identified promising subfamilies (clusters) for further research, and identified a new receptor-like kinase that specifically recognizes DspE and has properties distinct from those previously described.

## Conflict of interest

The authors declare no conflict of interest.
